# Methods for theoretical simulation of microvascular networks

**DOI:** 10.3389/fnetp.2026.1916707

**Published:** 2026-10-30

**Authors:** Timothy W. Secomb

**Affiliations:** Department of Physiology, University of Arizona, Tucson, AZ, United States

**Keywords:** angiogenesis, blood flow, flow regulation, microcirculation, network physiology, oxygen transport, structural adaptation, theoretical models

## Abstract

The microcirculation consists of an extensive network of small blood vessels. It is responsible for transport of substances including oxygen, nutrients and waste products to all parts of the body. Microcirculatory function can be studied experimentally using microscopic imaging techniques. However, many aspects of microvascular function are difficult to observe directly, and theoretical models and simulations have made valuable contributions to our understanding of the microcirculation. Here we review several theoretical approaches for investigating several key aspects of microvascular networks, including blood flow in microvessels, exchange of oxygen and other substances between blood and tissue, short-term local regulation of blood flow, and long-term structural adaptation and remodeling, including angiogenesis. A recurrent theme is the impact of the heterogeneous structure of the networks on the functional properties of the microvasculature. This heterogeneity arises from geometrical constraints and from the stochastic nature of microvessel growth. Theoretical simulations can be used to investigate how active mechanisms of flow regulation and remodeling can compensate for the inherent heterogeneity, and how failure of these mechanisms can contribute to impaired tissue function in diseases.

## 1 Introduction

All living organisms larger than a few cells in size require specialized mechanisms for the transport of metabolites and other substances throughout their tissues. In vertebrates, this function is largely served by a closed-loop system for the circulation of blood. The circulatory system can provide efficient transport of substances over relatively large distances by convection in the flowing blood. Other transport mechanisms, including passive molecular diffusion and active transport through cell membranes, are effective only over small distances, typically 100 μm or less. Therefore, the circulatory system must consist of a highly ramified branching system, in which relatively large vessels provide long-distance transport, while numerous tiny branches extend throughout metabolically active tissues. These branches, with diameters ranging from a few hundred microns to a few microns, constitute the microcirculation.

As a consequence of the small dimensions of microvessels, direct experimental investigation of microvascular function *in vivo* is challenging. Observations are limited by the spatial resolution of light microscopy, the limited depth of focus when imaging three-dimensional structures, and the difficulty of measuring solute concentrations non-invasively on small scales. Early imaging of microvascular network structures utilized thin sheet-like tissues including the mesentery (Poiseuille, 1835; Zweifach, 1954; Pries et al., 1990). Figure 1 shows an image of a network in the rat mesentery that was observed and mapped (Pries et al., 1990). Theoretical models and simulations provide methods to gain insight into biological processes occurring in microvascular networks, and for integrating and interpreting experimental observations. Data derived from imaging of network structure and blood flow in the mesentery has provided the basis for developing and testing some of these models.

**FIGURE 1:**
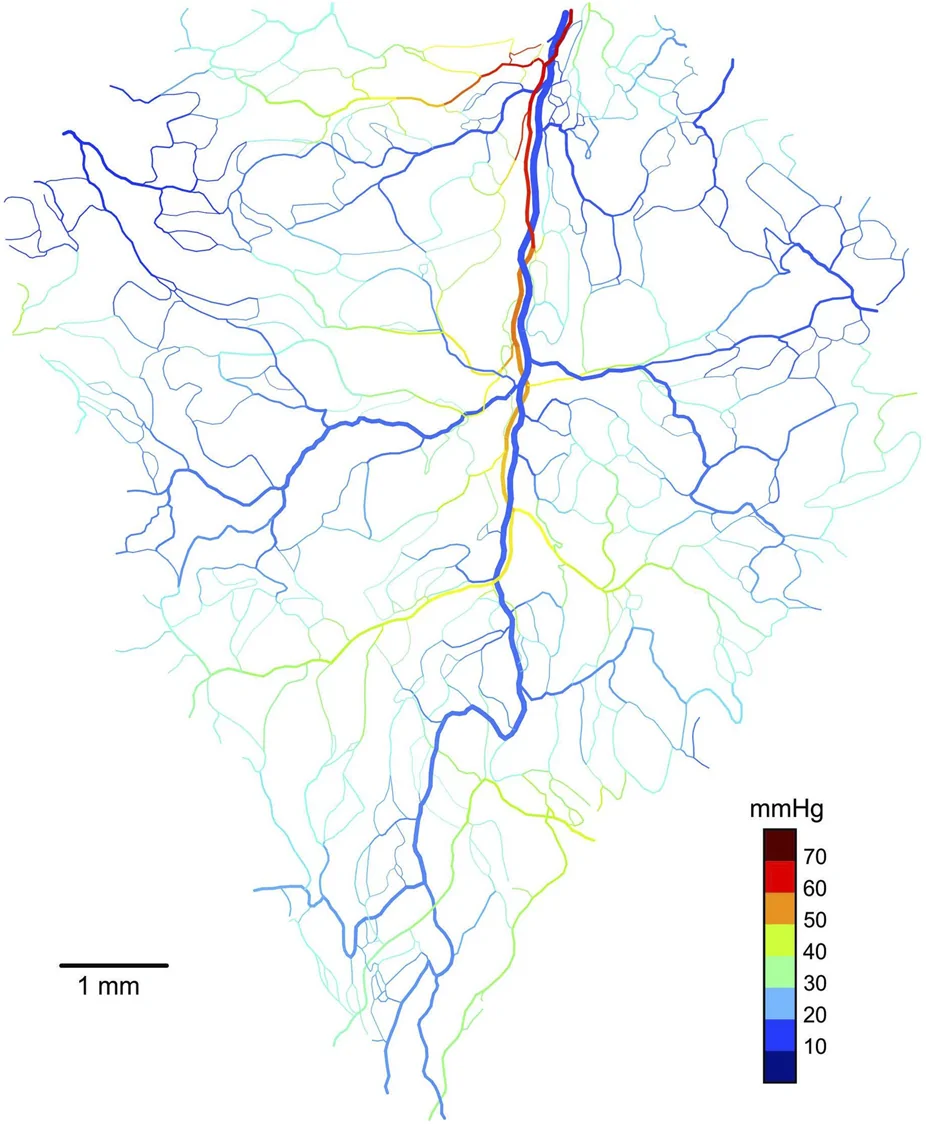
Microvascular network in the rat mesentery. This is “Network A″ with 913 segments as described by Pries et al. (1990). In this computer-generated image, the segments are color-coded according to local blood pressure, computed using the method described in Section 2.1.

In recent decades, technical advances in confocal and multi-photon microscopy have allowed the reconstruction of three-dimensional microvascular network structures from various tissues (Corliss et al., 2019), including brain (Gagnon et al., 2015), heart (Lee et al., 2009), tumor (Stamatelos et al., 2014) and retina (Milde et al., 2013). The availability of such data provides a basis for simulations based on observed network structures. This article presents a review of theoretical methods for simulating several key aspects of microcirculatory function from a network perspective.

## 2 Theoretical methods

### 2.1 Simulation of blood flow in microvessel networks

The methods used for simulating blood flow in the circulatory system vary considerably according to the diameter of the vessels under consideration. This can be understood in terms of the Reynolds number of the flow, which is defined as a typical ratio of the effects of fluid inertia to the effects of viscosity (Secomb, 2016). In arteries and veins, with diameters from about 1 mm up to several cm, the Reynolds number ranges up to 1,000 or more, inertial effects are highly significant, and flow patterns are highly dependent on the details of vessel geometry and on the variation with time of driving pressures.

In the microcirculation, by contrast, Reynolds numbers are generally much less than one, and inertial effects are negligible. To a good approximation, the flow in each vessel segment is then proportional to the instantaneous pressure drop along that segment, an important simplification. However, the analysis of blood flow in the microcirculation is complicated by non-continuum effects arising from the finite size of suspended elements, principally red blood cells. The diameter of a freely suspended human red blood cell is about 8 μm, which is comparable to the diameter of capillaries. The volume fraction of red blood cells in blood (hematocrit) is high, typically 40%–45%, and their effect on resistance to blood flow is highly dependent on both hematocrit and vessel diameter.

For a fluid with a fixed viscosity *μ*, the flow rate *Q* in a cylindrical tube with diameter *D* can be described by Poiseuille’s law:

$$Q=\frac{\pi }{128}\frac{\Delta pD^{4}}{L\mu }$$

where *L* is the tube length and *Δp* is the driving pressure. To describe the behavior of blood, it is convenient to define the apparent viscosity *μ*_*app*_ as the value of *μ* that satisfies this equation based on measured values of *Q* and *Δp*. Experiments using narrow glass tubes showed that this apparent viscosity decreases strongly with decreasing tube diameter over the range from about 300 μm down to about 10 μm.

This phenomenon, known as the Fåhraeus-Lindqvist effect, is a consequence of the mechanical deformability and physical dimensions of red blood cells, which causes behavior that is not adequately represented by simple continuum models. In narrow capillaries, red blood cells assume bullet-like shapes and flow in single file, with surrounding plasma forming a lubrication layer between the cells and the capillary wall (Secomb et al., 1986). In larger microvessels that allow multi-file flow, the cells tend to migrate away from vessel walls, forming a cell-free or cell-depleted layer (Secomb, 2017). Theoretical models have been developed to predict this behavior, based on cellular-level representation of red blood cell mechanics (Fedosov et al., 2010; Freund and Orescanin, 2011; Freund, 2014).

Observations of blood flow in microvessel networks in the rat mesentery showed that apparent viscosity *in vivo* is substantially higher than *in vitro* values (Pries et al., 1990). This difference was attributed to the presence of a layer of bound and adsorbed macromolecules, known as the glycocalyx or endothelial surface layer, that lines the walls of blood vessels (Pries et al., 2000). Empirical equations for the dependence of apparent viscosity *in vivo* on diameter and hematocrit (Pries et al., 1994; Pries and Secomb, 2005) are now generally used in simulations of microvessel hemodynamics.

A further consequence of the non-continuum behavior of blood in the microcirculation is the unequal partition of hematocrit in diverging microvessel bifurcations, leading to significant heterogeneity in microvessel hematocrit. Empirical equations (“bifurcation laws”) to describe the red cell flux fraction entering each branch of a bifurcation in terms of the diameters and flow rates of each branch and the hematocrit in the parent branch were developed based on observations in the mesentery (Pries et al., 1989; Pries and Secomb, 2005). These equations are often used in simulations of microvessel hemodynamics, although other formulations have been proposed. An alternative to these empirical equations is provided by cellular-level numerical simulation of blood flow in bifurcations within microvascular networks (Balogh and Bagchi, 2017).

The resistance to blood flow can be defined as the ratio of driving pressure to volume flow rate. If the apparent viscosity *μ*_*app*_ in a vessel segment is known, the segment’s flow resistance can be computed as

$$R=\frac{\Delta p}{Q}=\frac{128}{\pi }\frac{L\mu_{app}}{D^{4}}$$

The basic method for simulating flow in a network with known segment diameters, lengths and connectivity was first presented by Lipowsky and Zweifach (1974). The pressures at each interior node of the network are treated as the unknowns. The flows into each node are expressed in terms of the nodal pressures and the flow resistances, in a manner analogous to the analysis of electric circuits. The sum of the flows is set to zero, resulting in a set of linear equations for the pressures. Given boundary conditions, in the form of pressure or flow rate at each boundary node, this linear system can be solved by standard numerical methods, such as successive overrelaxation (Pries et al., 1990).

This method requires further development to allow for unequal hematocrit partition in diverging bifurcations, because flow resistance in each segment depends on hematocrit, which in turn depends on the flow distribution in upstream bifurcations. The hematocrit in all inflowing segments must be specified. An iterative method can be used (Pries et al., 1990). The steps are: (i) assign initial hematocrits in all segments; (ii) compute the apparent viscosity and the flow resistance of each segment according to the above equation; (iii) solve for the node pressures as already described; (iv) update the flows; (v) update the hematocrits using the bifurcation law; (vi) repeat steps (ii-v) until convergence of flows and hematocrits is achieved. Computer codes in C++ and sample input files are available online.^1^ For this and the other computer codes referenced throughout, the Supplementary Material contains descriptions of input and output files, definitions of associated parameters and other information.

The empirical equations as described above for flow properties of blood could eventually be replaced by results of cellular-level simulations. However, the physical properties of the endothelial surface layer have not so far been characterized with sufficient precision to allow reliable prediction of *in-vivo* apparent viscosity and hematocrit partition in bifurcations from first principles.

The method outlined above is applicable to networks in which the architecture is fully specified at the level of individual vessels including capillaries. In many situations of interest, actual capillary architecture is unknown due to limited resolution of imaging. Modeling approaches in such cases include generating a synthetic capillary network structure (Schmid et al., 2017) or treating the capillary network as a continuum (Peyrounette et al., 2018; Shipley et al., 2019).

As formulated above, the simulation of flow in networks neglects fluid exchange through the walls of blood vessels. In most tissues, the hydraulic conductivity of the endothelial cell layer is very low, such that total fluid filtration is well below 1% of the flow rate in capillaries, and can be neglected in calculating the distribution of intravascular flow. In that case, the intravascular pressure can be specified first, and then used to compute the net driving force for fluid filtration (Fleischman et al., 1986). If hydraulic conductivity is much higher, as in fenestrated capillaries or if permeability is increased as a result of inflammation, then fluid exchange may significantly affect intravascular flow (Rajeeva Pandian et al., 2026).

In most tissues, the microcirculation consists of a continuous mesh extending throughout the tissue. When a specific region is imaged, flow or pressure boundary conditions are generally not known for many small vessels that cross the boundaries of the region. To allow simulation of flow in such a microvascular network, a method was developed utilizing *a priori* information about typical levels of pressure and wall shear stress in microvessels (Fry et al., 2012). In this method, an objective function is defined as

$$D=\frac{1}{2}k_{p}\sum_{N}w_{k}{\left(p_{k}-p_{0k}\right)}^{2}+\frac{1}{2}k_{\tau }\sum_{S}l_{j}{\left(\tau_{j}-\tau_{0j}\right)}^{2}$$

where *N* is the set of internal nodes, *S* is the set of segments, *p*_0*k*_ is the target pressure at node *k*, *τ*_*j*_ is the wall shear stress in segment *j*, *τ*_0*j*_ is the corresponding target shear stress, *k*_*p*_ and *k*_*τ*_ are weighting factors associated with the pressure and shear deviations from the target values, and *w*_*k*_ is the vessel length associated with node *k*, defined as $w_{k}=\frac{1}{2}\sum l_{j}$ and the sum is over the segments *j* connected to node *k*. This objective function is minimized subject to constraints representing conservation of flow at each node, using the method of Lagrange multipliers. Because the flow directions in all segments are not known in advance, the dependence of the wall shear stress *τ*_*j*_ on the pressure difference in each segment is undetermined up to a change of sign. To avoid spurious solutions, the weighting factor *k*_*τ*_ is initially set to a small value and then successively increased, at each step using flow directions obtained in the previous step. For details, see Fry et al. (2012), and computer code is available.^2^

### 2.2 Oxygen transport

More than a century ago, a theoretical model was presented by August Krogh to describe the diffusion of oxygen from a capillary to a surrounding cylindrical region of tissue (Krogh, 1919). Subsequent development of models for oxygen transport was reviewed by Popel (1989) and Goldman (2008). To model oxygen transport to tissue by a network of vessels, the basic Krogh model must be extended to include the variation of oxygen levels in blood as it traverses the network, and the dependence of tissue oxygen levels on diffusion from multiple neighboring vessels, as discussed below.

Oxygen is a non-polar molecule and has similar ability to diffuse through both lipids and aqueous regions. The tissue can therefore be represented approximately as a homogeneous medium for oxygen diffusion. Convective transport of oxygen by interstitial fluid is generally negligible. Oxygen levels are conveniently represented in terms of the partial pressure (P_O2_), because this quantity is continuous across material boundaries. Under steady-state conditions, the tissue oxygen field satisfies

$$D\alpha \left(\frac{\partial^{2}P}{\partial x^{2}}+\frac{\partial^{2}P}{\partial y^{2}}+\frac{\partial^{2}P}{\partial z^{2}}\right)=M\left(P\right)$$ (1)

where *P* (*x*,*y*,*z*) is the P_O2_, *D* and *α* are the diffusivity and solubility of oxygen in tissue, and *M*(*P*) is the rate of oxygen consumption per unit volume. The dependence of consumption on P_O2_ is generally represented by Michaelis-Menten kinetics,

$$M\left(P\right)=\frac{M_{0}P}{P+P_{0}}$$ (2)

where *M*_0_ is the oxygen demand and *P*_0_ is the P_O2_ at half-maximal consumption rate.

Convective transport of oxygen by flowing blood is greatly enhanced by the binding of oxygen to hemoglobin. The rate of transport in an individual vessel is given by

$$J_{O2}=Q\left[C_{s}H_{D}S\left(P_{v}\right)+\alpha_{v}P_{v}\right]$$

where *Q* is the volume flow rate of blood in cm^3^/s, *C*_*s*_ ≈ 0.5 cm^3^O_2_/cm^3^ is the maximum oxygen carrying capacity per volume of red blood cells, *H*_*D*_ is the dimensionless (discharge) hematocrit where 0 < *H*_*D*_ < 1, *P*_*v*_ is the blood P_O2_ in mmHg, *S*(*P*_*v*_) gives the oxygen saturation of hemoglobin where 0 < *S* < 1, and *α*_*v*_ is the solubility of (unbound) oxygen in blood. The first term in this equation is normally dominant. The oxyhemoglobin saturation curve can be approximated by the Hill equation:

$$S\left(P_{v}\right)=\frac{P_{v}^{n}}{P_{v}^{n}+P_{50}^{n}}$$ (3)

where *P*_50_ and *n* are parameters that vary by species and are also affected by other variables including temperature and pH. If *q*(*s*) is the rate of oxygen efflux from the vessel per unit length, where *s* is distance along the vessel, then conservation of mass implies that

$$\frac{dJ_{O2}}{ds}=-q\left(s\right)$$

The solution of the above equations for oxygen transport from a network of vessels to surrounding tissue requires numerical methods. In principle, standard approaches such as finite-element and finite-difference methods could be applied. Unless the simulation region is small, however, these methods become impractical because very fine meshes are needed to resolve the small dimensions of microvessels and the steep gradients in oxygen levels around them. To address this challenge, an alternative approach was developed in which the vessels and tissue are treated as distributions of sources and sinks for oxygen diffusion, and the tissue oxygen field is expressed as a superposition of the fields resulting from each individual source or sink (the Green’s function) (Hsu and Secomb, 1989; Secomb et al., 2004). This approach takes advantage of the linearity of the Laplacian operator in Equation 1. However, a further challenge comes from the nonlinearity of Michaelis-Menten uptake kinetics and of oxygen binding to hemoglobin (Equations 2, 3), which necessitates the use of iterative schemes. A third challenge in this approach is the choice of appropriate boundary conditions. The apparently natural choice of zero-flux boundary conditions was found to lead to undesirable artifacts, and a preferable approach is to embed the simulation region in an infinite domain for the calculation of the Green’s function (Secomb et al., 2004). The Green’s function for a distributed source can then be approximated as that for a point source of the same total strength, which is inversely proportional to the distance from the source. A more detailed calculation is, however, needed for points very close to the source.

The basic steps in this method are: (i) compute the flow rate and hematocrit in each segment using the methods described in Section 2.1; (ii) subdivide the network into many short pieces; (iii) subdivide the tissue region into many small rectangular elements; (iv) express the complete oxygen field in terms of source strengths of vessel pieces and nodal points; (v) compute the source strength for each vessel piece and each nodal point so that vessel oxygen levels match the adjacent tissue levels at the blood-tissue boundary of each piece.

This method has been applied to simulate oxygen transport to tissue from networks of up to about 20,000 segments (Sharma and Secomb, 2025). A limitation of this approach is that it requires construction of a non-sparse N × N matrix, where N is the number of vessel network elements. A related method has recently been developed that avoids this limitation (Pastor-Alonso et al., 2024).

Implementations with and without graphical processor unit use are available online^3^ and additional information is provided in the Supplementary Material.

### 2.3 Local regulation of blood flow

The metabolic needs of all tissues in the body vary with time according to the functional state of the tissues. For example, both skeletal and cardiac muscle undergo wide variations in oxygen consumption between rest and exercise. Similarly, the transport activity of the gut increases after meals, and neuronal activity in the brain varies with different types of mental activity. The circulatory system includes elaborate mechanisms that provide local regulation of blood flow according to these changing needs, by varying the diameters of blood vessels and thereby altering their flow resistance.

When blood flows through a network of microvessels, the flow in any individual segment depends not only on the flow resistance of that segment, but also on the resistances of all the upstream and downstream segments that form a complete flow path through the circulation containing that particular segment. This has the important implication that an increase in metabolic demand in a particular region requires regulation of vessel diameters not only locally but also in remote vessels that supply the region. Of the total pressure drop between the arteries and the veins, more than 50% resides in the arterioles with diameters less than 100 μm (Pries et al., 1995), implying that the arterioles are a major site of flow resistance, and that significant increases in blood flow require dilation of these vessels. This is achieved by relaxation of the vascular smooth muscle cells that encircle arterioles. Upstream propagation of vasodilatory signals is therefore an essential component of local blood flow regulation (Segal, 2005). Such conducted responses are transmitted as electrical currents that pass between endothelial cells via gap junctions, which contain pores that connect adjacent cells. The network properties of the microcirculation are thus critical both for determining the distribution of blood flow and for the transmission of these conducted responses.

A review of theoretical models for regulation of blood flow up to 2008 was presented by Secomb (2008). Much of this work focused on models for responses of individual vessels to various signals. In the myogenic response, arterioles respond to an increase in intravascular pressure by contraction of vascular smooth muscle (Johnson, 1980). This is a key mechanism of flow autoregulation, the tendency of blood flow to remain nearly constant despite changes in arterial pressure. The shear-dependent response describes the dilation of arterioles in response to an increase in the shear stress acting on endothelial cells resulting from blood flow (Kuo et al., 1991). Metabolic responses are changes of vessel diameters in response to metabolite levels. Multiple mechanisms of metabolic flow regulation have been described, including responses to changes in levels of oxygen, carbon dioxide, potassium ions, adenosine and ATP. In muscle tissues, vasodilation in response to hypoxia plays an important role, although the mechanisms underlying this response remain the subject of debate (Kowalewska et al., 2024).

The studies referenced above did not explicitly consider the network structure of the microcirculation, instead for instance representing it as a segment with fixed flow resistance connected in series with an actively regulating arteriolar segment (Borgstrom and Grande, 1979). A step towards more explicit representation of network properties is the “representative segment model” (Arciero et al., 2008; Carlson et al., 2008). In this approach, the systemic vasculature is represented by seven compartments connected in series: arteries, large arterioles, small arterioles, capillaries, small venules, large venules, veins (Figure 2). Within each compartment, a number of equivalent segments are connected in parallel, so that the flow rate in that segment is appropriate to the vessel diameter. The large and small arterioles are vasoactive, and the remaining segments are fixed resistances.

**FIGURE 2:**
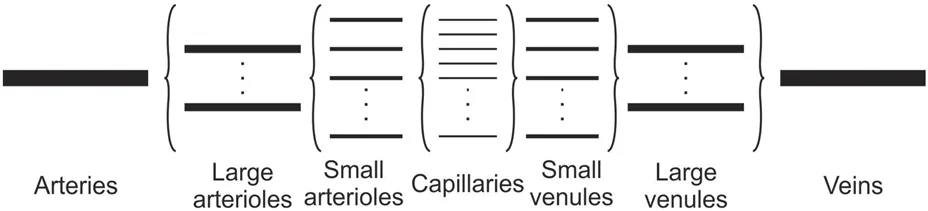
Representative segment model for a microvascular network. All segments within each compartment are treated as equivalent. Segment lengths and diameters are not shown to scale.

A versatile framework for modeling active flow regulation by arterioles was developed (Arciero et al., 2008; Carlson et al., 2008). In this framework, a quantity *S*_*tone*_ is defined as the sum of the vasoactive signals under consideration, which may include myogenic, shear dependent, metabolic and conducted signals. The target activation of vascular smooth muscle is calculated as

$$A_{total}=\frac{1}{1+\exp\left(-S_{tone}\right)}$$

where the sigmoidal dependence on *S*_*tone*_ ensures that 0 < *A*_*total*_ < 1. Here, *A*_*total*_ = 0 represents complete relaxation and *A*_*total*_ = 1 represents maximal activation of the muscle. The dynamics of tone development are represented by

$$\frac{dA}{dt}=\frac{1}{\tau_{a}}\left(A_{total}-A\right)$$

where *A* represents the actual activation, and *τ*_*a*_ is a time constant. The wall tension is the sum of active and passive components

$$T_{total}=T_{pass}+AT_{act}^{\max}$$

where *T*_*pass*_ represents the passive tension in a vessel wall and is given by an exponential function of diameter, and $T_{act}^{\max}$ represents the maximal active tension and is defined by a Gaussian function of diameter. Changes in diameter *D* are driven by a mismatch between this wall tension and the tension required to balance intravascular pressure *P*, which is given by the law of Laplace, T=PD/2 :

$$\frac{dD}{dt}=\frac{1}{\tau_{d}}\frac{D_{c}}{T_{c}}\left({T-T}_{total}\right)$$

where *D*_*c*_ and *T*_*c*_ are reference values of diameter and tension, and *τ*_*d*_ is a time constant.

This modeling approach was applied to investigate the contributions of the various mechanisms of flow regulation to the control of blood flow in skeletal muscle (Arciero et al., 2008; Carlson et al., 2008). With regard to autoregulation of blood flow, it was found that both myogenic and metabolic responses must be present to account for the observed maintenance of blood flow in the face of changes of arterial pressure. As expected, the modulation of blood flow in response to changes in oxygen demand was shown to depend on the presence of a metabolic response and its upstream propagation as a conducted response. An unexpected finding was that both the myogenic and shear-dependent responses had the effect of slightly reducing blood flow at high levels of oxygen consumption.

The representative segment model effectively assumes that the microvascular network has a completely symmetric branching pattern. In reality, the network structures are heterogeneous in structure, with wide variations in segment lengths within each category of vessels, and unequal flow partition at diverging bifurcations. The effects of this asymmetry in the context of flow regulation have been examined theoretically using the above framework to represent the active responses of arteriolar segments (Fry et al., 2013). In this case, the three innermost compartments in the representative segment model are replaced by a microvascular network observed in the hamster cremaster muscle (Berg et al., 1997), as shown in Figure 3. The results provide an explanation for the observed phenomenon of sequential recruitment of individual capillaries in response to increases in metabolic demand.

**FIGURE 3:**
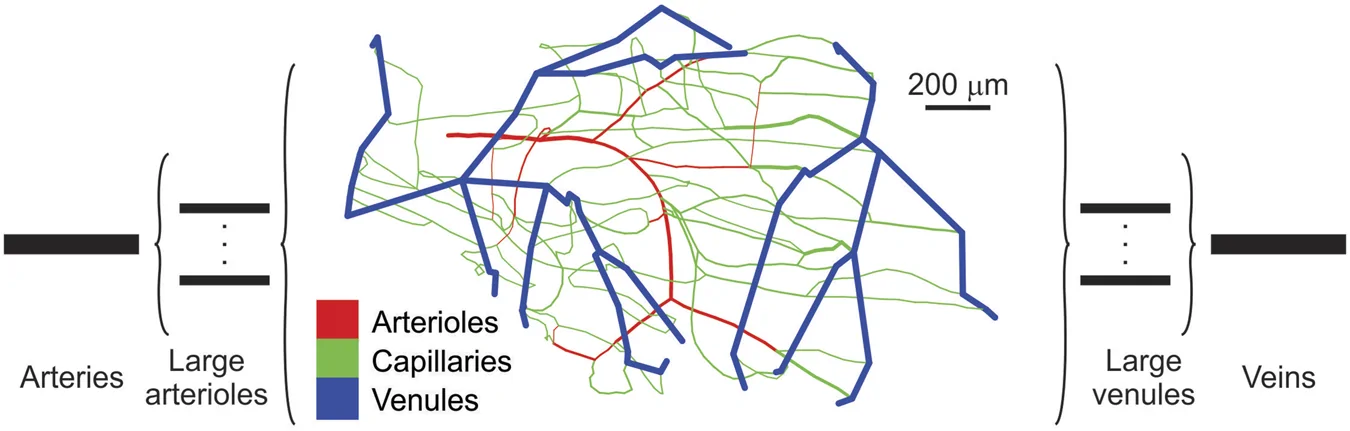
Model used to examine effects of asymmetric network structure on metabolic flow regulation (Fry et al., 2013). A microvascular network observed in the hamster cremaster muscle is connected in series with four compartments of the representative segment model shown in Figure 2, to form complete flow pathways. In the computer-generated network image, segments are color-coded according to type (arteriole, capillary, venule).

### 2.4 Structural adaptation of vessel diameters

As discussed in Section 2.3, short-term regulation of blood flow is achieved primarily by active contraction of vascular smooth muscle in arterioles. The vascular system is also capable of longer-term adjustments to functional requirements, by structural adaptation of vascular diameters and by changes in the number of blood vessels.

The signals that drive structural adaptation parallel those that cause short-term changes in vascular smooth muscle tone. Increases in wall shear stress drive outward remodeling of vessel walls, while increases in pressure drive wall thickening and a decrease in luminal diameter. Increased metabolic stimuli cause increase in vessel diameter, and upstream conducted responses are essential in coordinating diameter changes along flow pathways (Pries et al., 1998). These parallels between short and long-term diameter changes are consistent with observations that sustained changes in vascular smooth muscle tone cause corresponding structural adaptation of vessel diameters (Bakker et al., 2000; Martinez-Lemus et al., 2009).

Early development of models for structural adaptation of vessel diameters focused on responses to wall shear stress (Rodbard, 1975; Kamiya et al., 1984). It was proposed that vessels adjust their diameters *D* so as to maintain a fixed or target level (*τ*_0_) of wall shear stress *τ*:

$$\frac{1}{D}\frac{dD}{dt}=k\left(\tau -\tau_{0}\right)$$ (4)

where

$$\tau =\frac{32}{\pi }\frac{\mu_{app}Q}{D^{3}}$$

Here, *k* is a constant and *Q* is volume flow rate. In this model, a wall shear stress above the target level leads to increase in diameter, which restores stress to the target value, if a fixed flow rate is assumed. According to the theory proposed in 1926 b y Murray (Murray, 1926; Kamiya et al., 1984), such maintenance of wall shear stress at a target level results in an optimal balance between the energy cost of pumping blood and the cost of maintaining a given volume of blood.

An observed feature of vascular beds is that venous vessels are typically larger than corresponding arterial vessels carrying the same blood flow rates. This effect can be incorporated into the above model by assuming that the target shear stress is an increasing function of intravascular pressure (Pries et al., 1995). The resulting long-term structural adaptation is analogous to the acute myogenic response.

However, when this model is applied in the context of vessel networks, unrealistic behavior is found (Hudetz and Kiani, 1992; Hacking et al., 1996). For example, if two segments of equal length are connected in parallel, then the segment with larger diameter experiences higher flow and higher wall shear stress, and therefore enlarges, while the other segment shrinks. According to this model, any network with a single inlet and outlet would eventually decay to a single flow pathway, which is clearly not the case in microvascular networks.

This apparent contradiction can be resolved within the model by recognizing that microvascular networks can meet metabolic needs of the tissue only if the incoming flow is divided among numerous small branches, and by including a term in the model in Equation 4 that represents a response to this requirement. Models were developed that include a growth term that increases at low vessel flow rates or at low local oxygen levels. These models exhibited stabilization of multiple parallel flow pathways (Pries et al., 1998; Pries et al., 2001).

Even these models including metabolic response terms were, however, found to be inadequate to describe the distributions of vessel diameters and flow rates observed in mesenteric microvascular networks (Pries et al., 1998). The discrepancies were related to the heterogeneous branching patterns of the networks, and particularly to the existence of short and long flow pathways connecting input and output vessels. Segments within short flow pathways tend to experience high wall shear stress and to enlarge, resulting in low flow resistance and diverting flow away from longer flow pathways. Such behavior, termed “functional shunting,” is not generally seen in normal healthy tissue.

Further elaboration of the model was needed to address this deficiency (Pries et al., 1998). Each segment was assumed to respond not only to purely local signals but also to signals propagated from distal to proximal segments, such that proximal segments supplying or draining more extensive distal networks receive a larger growth signal. For proximal feeding segments, this signal must travel in opposite direction to the blood flow. Such upstream propagation is assumed to be achieved by conducted responses in the vessel wall, as discussed in Section 2.3. Downstream signal propagation can occur by convective transport of metabolites.

Models including responses to all four abovementioned types of stimulus, i.e., wall shear stress, intravascular pressure (or circumferential wall stress), local metabolic conditions, and upstream and downstream propagated signals, appear to be of minimal complexity to describe structural adaptation in microvascular networks (Pries et al., 1998). In particular, the contribution of upstream conducted responses to structural adaptation of vessel diameters appears to be essential for the maintenance of functionally adequate microvascular networks. Loss or reduction of conducted responses is a likely explanation for aberrant vascular network structures seen in the microcirculation of solid tumors (Pries et al., 2010).

### 2.5 Angiogenesis and pruning

The structural response of the microcirculation to changing demands requires not only adjustments of segment diameters but also addition of vessel segments (angiogenesis) or their removal (pruning) (Risau, 1997; Pries and Secomb, 2014). Four basic modes of angiogenesis have been identified: vasculogenesis, sprouting angiogenesis, intussusceptive (splitting) angiogenesis, and coalescent angiogenesis. Vasculogenesis refers to the early embryonic formation of a vascular plexus from precursor vascular cells (Risau and Flamme, 1995). In sprouting angiogenesis, endothelial cells form new branches from existing vessels. These branches elongate and eventually connect with other vessels to form closed flow pathways (Risau, 1997). Intussusception refers to the splitting of the lumen along the length of an already flowing vessel, so that two or more parallel lumens are formed (Risau, 1997). Blood flow is maintained throughout this process, in contrast to sprouting where sprout formation precedes initiation of blood flow in the sprout. Identified more recently, coalescent angiogenesis refers to the merging of multiple adjacent vessels into a single vessel with a larger lumen (Nitzsche et al., 2022). This is the inverse process to splitting angiogenesis, and leads to apparently equivalent structures at intermediate time points, making it difficult to distinguish from intussusception.

Theoretical modeling of network formation by angiogenesis has generally focused on sprouting angiogenesis, particularly in the context of tumors or wound healing (Anderson and Chaplain, 1998; Hahnfeldt et al., 1999; Levine et al., 2001; Tong and Yuan, 2001; Owen et al., 2009; Welter and Rieger, 2010; Perfahl et al., 2011; Vilanova et al., 2017; Fredrich et al., 2018; Flegg et al., 2020). Models for other modes of angiogenesis have received less attention. A number of methods have been used, including both continuum and discrete approaches, as reviewed by Scianna et al. (2013).

In models for sprouting angiogenesis, sprout growth is generally assumed to be controlled by concentrations of diffusible substances, including metabolites and growth factors. Continuum models are appropriate for describing substrate diffusion. However, network structure is more readily described in discrete terms (segments and nodes). Thus, hybrid models combining continuum and discrete approaches are well suited for simulation of sprouting angiogenesis, and have been used in a number of the studies cited above.

In a series of studies, a hybrid model of sprouting angiogenesis was combined with the model for structural adaptation described in Section 2.4, and used to simulate angiogenesis in mesentery, brain and retina (Secomb et al., 2013; Alberding and Secomb, 2021; 2023). In the resulting model, once flow is established in a given segment, its diameter is treated as a dynamic variable that changes in response to both hemodynamic signals (wall shear stress and internal pressure) and metabolic signals dependent on oxygen levels in adjacent tissue. The model includes effects of upstream and downstream propagated metabolic signals. In this approach, pruning of redundant segments is automatically represented. If a segment does not experience significant levels of either wall shear stress or metabolic stimulus, then its diameter decays below a level that can support blood flow and it is pruned. An example of a simulated network structure in the brain cortex is shown in Figure 4, and computer code is available online.^4^

**FIGURE 4:**
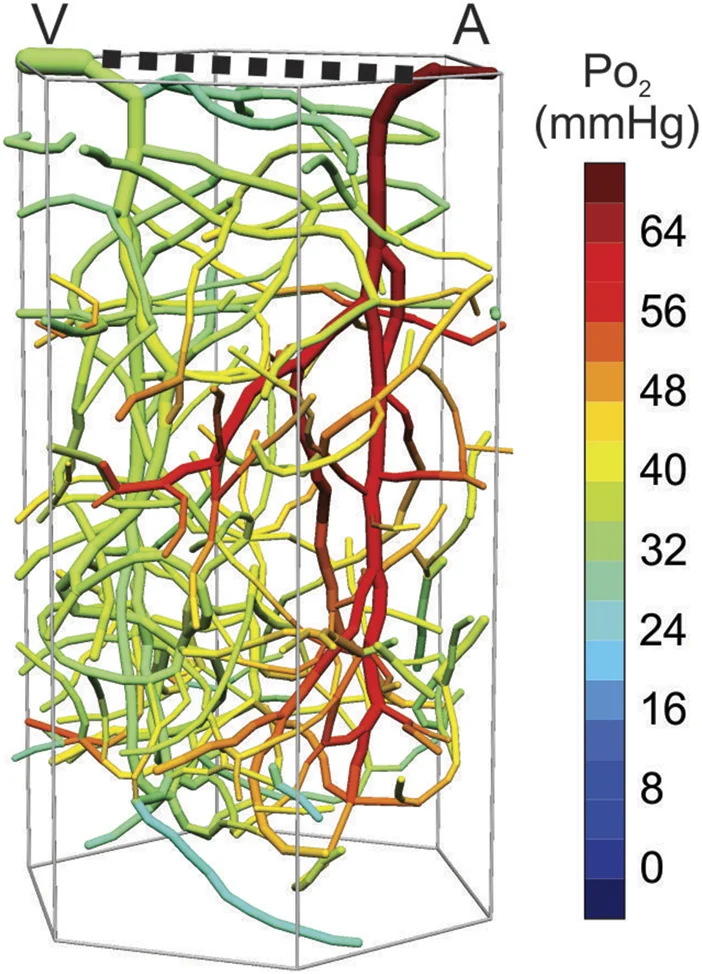
Example of a vascular network generated by a model for sprouting angiogenesis and structural adaptation in the brain cortex (Alberding and Secomb, 2021). Dimensions of region are approximately 300 × 300 × 500 μm. Partial pressures of oxygen in blood are indicated by colour bar. A periodic array of such hexagonal units forms a continuous capillary mesh representing the cortical microcirculation. Each vessel segment that enters one side of the hexagonal unit has a corresponding segment that exits on the opposite side, except for the feeding arteriole (A) and the draining venule (V). The dotted line indicates the initial position of the pial vessel from which the network is formed by sprouting. This connection regresses and is pruned during the simulated angiogenesis.

Simulations using this approach confirm the importance of upstream conducted responses for the formation of functionally adequate network structures. For example, the vascular system of the brain cortex is formed during embryonic development by vessels that branch off an initial plexus of pial vessels on the surface. In the process, arterio-venous connections that are initially present in the pial network must regress, otherwise they would form functional shunts, and blood flow would bypass the deeper regions of the cortex. Simulations with compromised conducted responses show such behavior, resulting in hypoxia within the cortex (Alberding and Secomb, 2021).

## 3 Discussion

The circulatory system of a human contains vessels with a total length of order 10^7^ m (Poole et al., 2021). Most of the vessels are capillaries with lengths below 1 mm, implying that the total number of microvessels is of order 10^10^ or more. Evidently, the location and size of each individual segment are not under direct genetic control. Instead, network structures must emerge as a result of generic responses of each segment to the various stimuli that it experiences (Secomb et al., 2012). In principle, experimental techniques of molecular and cellular biology can be used to identify and understand the mechanisms underlying these responses, but progress toward this goal is far from complete.

In this context, theoretical models can play a valuable role, by identifying the types of vascular responses that are necessary and/or sufficient to account for observed network-level behaviors of the vasculature, and showing how they can be combined to achieve such behaviors. From this perspective, a theoretical model of a biological system is a test of a hypothesis, namely, the set of assumptions and parameter values on which the simulation is based. Comparisons between predicted behavior and experimental observations provide tests of the hypothesis. Agreement between predicted and observed behavior supports the hypothesis, but does not prove the validity of the model. On the other hand, lack of agreement can imply the need to re-examine the assumptions of the model and can lead to improved understanding of the system.

The models described in the preceding sections provide examples of this approach. When the network flow model was initially applied to the rat mesentery, the predictions did not agree with observed characteristics (Pries et al., 1990). This discrepancy led to the recognition that the endothelial surface layer has a substantial effect on flow resistance in microvessels. Similarly, predictions of initial models for structural adaptation of vessel diameters were inconsistent with observed structures. This discrepancy was resolved by including the effects of conducted responses in the control of network structure (Pries et al., 1998), a phenomenon for which direct experimental evidence has been slow to emerge (Figueroa et al., 2006; Buschmann et al., 2010; Pohl, 2020).

The typically heterogeneous structure of microvascular networks is immediately evident on observation (Figure 1). Several causes for this heterogeneity can be identified (Pries and Secomb, 2009; Roy and Secomb, 2021). Firstly, angiogenesis is a stochastic process that leads to heterogeneous structures. Secondly, feeding and draining vessels with tree-like branching structures are combined with a mesh-like capillary network that must encompass the entire tissue region, and this inevitably leads to heterogeneity. Thirdly, major feeding and draining vessels often lie adjacent to each other, with the consequence that flow pathways connecting them vary greatly in length. In this context, the processes of flow regulation, structural adaptation and angiogenesis as described above can be viewed as compensating for this inherent heterogeneity, in order to achieve adequate and efficient distribution of oxygen and nutrients to tissue.

The role of upstream conducted responses in directing flow into longer flow pathways and avoiding functional shunting by short flow pathways is an example of such a mechanism. These conducted responses are critically dependent on the proper functioning of gap junctions that connect adjacent endothelial cells in vessel walls (Djurich et al., 2025). Loss of conducted responses can lead to maldistribution of blood flow and to tissue hypoxia or anoxia (Roy and Secomb, 2021; 2022). During inflammatory responses, gap junction communication is typically impaired (Baker et al., 2008). According to these arguments, disorders including coronary microvascular disease (Taqueti and Di Carli, 2018) and cerebral small vessel disease (Markus and Joutel, 2025) may result from failure of mechanisms responsible for mitigating the inherent heterogeneity of the microvasculature. More broadly, these arguments imply that the network physiology of the microcirculation is a key determinant of circulatory system function.

Current experimental research in vascular biology largely emphasizes molecular and cellular-level processes. However, the functional behavior of the vasculature in living tissue depends on the integrated, network-level consequences of these processes. Given the technical difficulty of direct observations of microcirculatory phenomena *in vivo*, theoretical models such as those described here will continue to be useful in gaining understanding of vascular function. For example, when potential mechanisms of metabolic blood flow regulation are identified, the modeling framework described in Section 2.3 can be adapted to test whether such mechanisms can generate responses consistent with observed behavior. The effects of vasoactive agents on heterogeneity of blood flow and tissue oxygenation could be explored using such models, with potentially significant implications for the management of sepsis among other conditions. Similarly, the model described in Section 2.4 could be used as a testbed for examining the functional effects of newly identified molecular and cellular processes in vascular remodeling, and the effects of agents that affect these processes. The models presented here must be regarded as works in progress, likely requiring further development to address such topics.

## Data Availability

The raw data supporting the conclusions of this article will be made available by the authors, without undue reservation.

## Footnotes

1
https://github.com/secomb/NetFlowV2

2
https://github.com/secomb/FlowEstimateV1

3
https://github.com/secomb/GreensV4, https://github.com/secomb/GreensV4_GPU

4
https://github.com/secomb/AngioAdapt20
