# Supplementary material for: Methods for theoretical simulation of microvascular networks
Source: Front Netw Physiol. 2026 Oct 30;6:1916707. doi: 10.3389/fnetp.2026.1916707 (PMC13759275; doi:10.3389/fnetp.2026.1916707)
Supplement: Supplementary file 1 [file DataSheet1.pdf]

Supplementary Material

Theoretical simulation of microvascular networks

Timothy W. Secomb

Department of Physiology, University of Arizona, Tucson, Arizona, USA

README files for computer codes available on GitHub

1. NetFlowV2: Simulation of microvascular network hemodynamics

2. FlowEstimateV1: Estimation of blood flows in a network with incomplete boundary conditions

3. GreensV4_GPU: Simulation of solute transport in vascular networks (GPU version)

4. AngioAdaptRetina: Simulation of angiogenesis, remodeling and pruning in the retina

1. NetFlowV2: Simulation of microvascular network hemodynamics

https://github.com/secomb/NetFlowV2

This program is designed to simulate the dynamics of blood flow in networks of microvessels. Such simulations are a necessary component in theoretical models for several aspects of the microcirculation, including transport of oxygen and other solutes [1], structural adaptation of microvessels [2–4], and regulation of blood flow [5].

The simulation is based on a series of studies, in which we developed empirical equations to describe the dynamics of blood flow in microvascular networks. The key components are:

- equations to describe the apparent viscosity of blood in microvessels in vivo as a function of vessel diameter and discharge hematocrit [6,7];
- equations to describe the partition of hematocrit in diverging bifurcations, as a function of the flow rates in the branches, the diameters of the branches, and the hematocrit arriving at the parent vessel [7,8].

The parameters describing these relationships are given in RheolParams.dat. This gives parameters needed for estimating the apparent viscosity in each segment. Set varyviscosity = 1 to get diameter-dependent viscosity. Set phaseseparation = 1 to compute phase separation in diverging bifurcations. This can lead to nonconvergence of the method in some cases. Also, the number of iterations for linear and nonlinear loops are specified. These may need to be increased for very large networks.

The equations used in the simulations are the versions given in our article in the Handbook of Physiology [9]. These equations allow the prediction of flow rates, pressures and hematocrits throughout the network, provided that the following information is available:

- the network topology;
- the lengths and diameters of each segment;
- the boundary conditions at every boundary segment (i.e. segment flowing in or out of the network), in the form of specified pressure or flow, including at least one pressure boundary condition;
- specified hematocrits at each inflowing boundary segment.

In the implementation provided here, it is assumed that the spatial coordinates of each node are known. The length of each segment is computed as a straight-line distance. Curved segments can be approximated by combining several straight segments.

The above information is conveniently represented in a file typically named network.dat (see example). On output, the file is renamed NetworkNew.dat, and contains the computed flow and hematocrit values for each segment. This file consists of four parts:

- header and global parameters. Except for nseg (number of segments) these parameters are ignored by NetFlow; they are used for simulation of solute transport (see GreensV4).
- list of segments with the following details for each segment:
    - name: integer, not necessarily sequential
    - type: integer, if type is not 4 or 5, the segment is ignored

  - from: integer, 'from' node
  - to: integer, 'to' node
  - diam.: real, diameter in microns
  - flow: flow in nl/min.: On input, the value is ignored. On output, the computed value is given
  - hem.: hematocrit from 0 to 1.: On input, the value is ignored. On output, the computed value is given
- list of nodes with the following details for each node:
  - name: integer, not necessarily sequential
  - x,y,z: Cartesian coordinates of node in micron
- list of boundary nodes with the following details for each node:
  - name: integer, as in node list
  - bctyp: integer, 0 for defined pressure, 2 for defined inflow rate
  - press/flow: boundary pressure in mmHg or flow in nl/min
  - HD: inflow discharge hematocrit. Ignored for outflow nodes
  - PO2, solute 3, etc.: solute data, ignored by this program

The program generates several postscript files:

- histograms of node pressure (mmHg), segment flow velocities (mm/s), segment flow rates (nl/min) and shear stress (dyn/cm^2)
- network geometry visualized as projected on a plane. The position and orientation of the plane are specified by ContourParams.dat, giving the coordinates of three corners. Each vessel segment is color-coded according to midpoint pressure.

The program generates greens.exelem and greens.exnode, allowing 3D visualization of the network using cmgui. When cmgui is started, use File: Open: com file, select greens.com.txt and hit “All” for the visualization. For details of cmgui, see:
http://sourceforge.net/projects/cmiss/files/cmgui/cmguiwx2.8.0/

This code is free to use at your own risk. Feedback and/or acknowledgement are appreciated. Email secomb@u.arizona.edu.

Note: This code makes use of nrutil.h and nrutil.cpp as placed in the public domain by Numerical Recipes at http://apps.nrbook.com/c/index.html.

1. Secomb TW, Hsu R, Park EY, Dewhirst MW (2004) Green's function methods for analysis of oxygen delivery to tissue by microvascular networks. Ann Biomed Eng 32: 15191529.
2. Pries AR, Secomb TW, Gaehtgens P (1998) Structural adaptation and stability of microvascular networks: theory and simulations. Am J Physiol 275: H349H360.
3. Pries AR, Reglin B, Secomb TW (2001) Structural adaptation of microvascular networks: functional roles of adaptive responses. Am J Physiol Heart Circ Physiol 281: H1015H1025.
4. Pries AR, Reglin B, Secomb TW (2005) Remodeling of blood vessels: responses of diameter and wall thickness to hemodynamic and metabolic stimuli. Hypertension 46: 725731.
5. Roy TK, Pries AR, Secomb TW (2012) Theoretical comparison of wall-derived and erythrocyte-derived mechanisms for metabolic flow regulation in heterogeneous microvascular networks. Am J Physiol Heart Circ Physiol 302: H1945H1952.

6. Pries AR, Secomb TW, Gessner T, Sperandio MB, Gross JF, Gaehtgens P (1994) Resistance to blood flow in microvessels in vivo. Circ Res 75: 904915.
7. Pries AR, Secomb TW (2005) Microvascular blood viscosity in vivo and the endothelial surface layer. Am J Physiol Heart Circ Physiol 289: H2657H2664.
8. Pries AR, Ley K, Claassen M, Gaehtgens P (1989) Red cell distribution at microvascular bifurcations. Microvasc Res 38: 81101.
9. Pries AR, Secomb TW (2008) Blood Flow in Microvascular Networks. In: Tuma RF, Duran WN, Ley K, editors. Handbook of Physiology: Microcirculation. Second Edition. San Diego: Academic Press. pp. 336.

2. FlowEstimateV1: Estimation of blood flows in a network with incomplete boundary conditions

https://github.com/secomb/FlowEstimateV1

The purpose of this program is to estimate blood flows in a network of microvessels with known geometry, but with incomplete boundary conditions. If a complete set of boundary conditions is given, then NetFlowV1 can be used: see https://github.com/secomb/NetFlowV2.

The essential idea of the method is to solve for a set of flows that satisfies conservation of flow at all internal nodes and also satisfies any known boundary conditions, while minimizing the sum of squared deviations of pressures and flows from target values based on typical hemodynamic properties of such networks. The method is described in:
Fry BC, Lee J, Smith NP, Secomb TW. 2012 Estimation of blood flow rates in large microvascular networks. Microcirculation 19, 530-538. (doi:10.1111/j.1549-8719.2012.00184.x).

An objective function is set up in terms of the unknown nodal pressures. The constraints of flow conservation at internal and known boundary nodes are introduced using Lagrange multipliers. Differentiating with respect to the pressures and the Lagrange multipliers gives a system of linear equations:

```
              N                      IUB'
 __                                        __  __  __       __                __
 |                        |                 |  | p1 |       |                  |
 |                                          |  | p2 |       |                  |
 |    H' = 1 + ktH/kpw    |       -K^T      |  | p3 |       |p+kt*LMtau/kpw    |
 |          or 1                            |  | .  |       |   or fixed p     | N
 |                        |                 |  | .  |       |                  |
 |                                          |  | pn |       |                  |
 |-----------------------+-----------------|  |----|   =   |------------------|
 |                                          |  | l1 |       |       q01        |
 |                        |                 |  | l2 |       |       q02        |
 |                                          |  | l3 |       |       q03        |
 |           -K           |        0        |  | .  |       |  also includes   | IUB'
 |                                          |  | .  |       |   terms from     |
 |                        |                 |  | .  |       | fixed p values   |
 |                                          |  | .  |       |        .         |
 |_                       |               __|  |_ __|       |_              __|
```

where N = nnod = total number of nodes;
Nkp = numberknownpress = number of boundary nodes with known pressures;
Nu = numberunknown = number of boundary nodes with unknown pressures and flows;
IUB' = number of internal nodes + number of boundary nodes with known flow = N - Nu - Nkp;
IUB' + N = matrixdim = 2*N - Nkp - Nu is the dimension of the matrix.

The target shear stress refers only to the magnitude and the flow directions are unknown a priori. This introduces a combinatorial aspect into the optimization problem. A heuristic approach is used. The parameter ktau, which gives the weight of the squared shear stress deviations in the objective function, is increased stepwise, with the target flow direction set in each segment set

equal to the computed flow direction at the previous step. The outline of the procedure is: (1) Start with ktau = 0. (2) Determine flows. (3) If a segment flow is negative, set the corresponding flow direction to -1, so the target shear stress is set to be negative. (4) Repeat until flow directions no longer change for the given ktau value. (5) Successively double ktau, and repeat (2), (3) and (4). Flows are given in nl/min, pressures in mmHg, viscosities in cP, lengths and diameters in microns and times in s. In these units, the procedure is repeated until the ratio of ktau to kpress (the pressure deviation weight) is 4.096, as found by Fry et al. (2012).

The original implementations of the method used a direct method (LU decomposition) to solve the linear system. For large networks (thousands of nodes) this method is slow and may run into memory limitations. The current version provides a choice of a direct solver or an iterative method that takes advantage of the sparseness of the system. The system is arranged so as to make it strictly symmetric and a preconditioner is used to put ones on the diagonals of the H' and K matrices. Then the conjugate gradient method is used to solve the system.

Notes

We supply several sample problems in the sub-directories. For input file formatting, please see the files in these directories. These files contain further explanatory comments.

1. Input files must be placed in the same working directory where the C code resides. They can be copied from the folders containing sample problems. The following input files are used: Network.dat This file specifies the network structure, and the known boundary conditions. It uses the same structure as Network.dat files for NetFlow and Greens, but with some significant differences. (1) The data on lines 1 to 6 of the file are ignored. (2) The flow rates and hematocrits listed for each segment are ignored. However, the sign of the flow rates in this section are used to compute the number of segments with reversed flows (relative to the given data). (3) In the final section, boundary conditions are included only for boundary nodes where the boundary pressure (type 0) or flow (type 2) is given. ContourParams.dat Although no contour plots are generated, the coordinates of three corners are used to define the plane on which the network is projected in picturenetwork.cpp. RheolParams.dat This gives parameters needed for estimating the apparent viscosity in each segment. Set varyviscosity = 1 to get diameter-dependent viscosity. Set phaseseparation = 1 to compute phase separation in diverging bifurcations. Warning: including this effect leads to unstable flow directions and lack of convergence of the method. It is preferable to assume fixed viscosity. FlowEstParams.dat These values are specific to this program: target pressure, target shear stress, number of ktau steps, maximum number of iterations for each tau value.

2. The program generates several output files: Run_summary.out tracks the variation in RMS deviations of pressure and shear stress from target values Results.out gives statistics of the resulting distributions of variables, and a list of estimated variables for each segment. NetworkNew.dat recreates the Network.dat file but with the computed flow values (and possibly hematocrits). The list of boundary nodes is now complete, and is given in terms of pressure boundary conditions. histogram-logflows.out.ps, histogram-logstress.out.ps and histogram-pressures.out.ps give histograms of the data in the corresponding .out files, for log10(flow), log10(shear stress) and pressure. network.exelem, network.exenode and cmgui.com.txt can be

used to obtain 3D visualizations of the network using CMGUI. When CMGUI is started, use File - Open - com file, select greens.com.txt and hit “All” for the visualization. For details of cmgui, see: http://sourceforge.net/projects/cmiss/files/cmgui/cmgui-wx-2.8.0/

3. An option has been added to specify flow direction in boundary segments without specifying flow rate. This is done by setting bctyp[inodbc] = -2 in the boundary conditions section of network.dat. A fixed weight (known_flow_weight) is placed on that segment in the objective function. Note that the flow could possibly still be reversed from the specified direction.

4. Three choices of linear solver are specified by the parameter solvetyp in FlowEstParams.dat:
- solvetyp = 1: LU decomposition, suitable for small networks, up to about 1000 nodes.
- solvetyp = 2: Sparse conjugate gradient method. This method is robust but slow for very large networks (e.g. 25000 nodes).
- solvetyp = 3: Successive over-relaxation. This method can be faster than method 2 in some cases, but requires careful optimization of relaxation parameters nitmax3 and omega2. If omega2 is too large, the iteration diverges. If omega2 is too small, convergence is slow. The best range for nitmax3 seems to be around 40, but this may depend on network properties.

5. A factor kappa is introduced multiplying the target shear stress values in the objective function, where kappa = mean(tau^2)/(mean(tau))^2. This is needed to compensate for bias in least squares estimation. Otherwise, estimated tau values are biased to be small, because this reduces the variance in tau.

6. On output, all boundary segments are classified according to the following scheme: 9: Inflow arteriole 8: Outflow arteriole 7: Inflow capillary 6: Outflow capillary 5: Inflow venule 4: Outflow venule. This classification is based on (1) inflow or outflow; (2) diameter compared to diamcrit; (3) pressure compared to mean capillary pressure.

7. A routine flowtest() is applied to the network before flow estimation. This routine tests for segments that have identically zero flow (e.g. floating loops) and removes them from the network, along with associated nodes. Such segments can cause singularity of the linear system being solved. Note that the segments and nodes are renumbered as part of this procedure.

8. We have tested this package using Microsoft Visual C++ 2017 under Windows and linux. For linux, a makefile is included: see the instructions in the file and remove the linux.txt extension before use. For error reporting and suggestions please contact Dr. Timothy W. Secomb, email secomb@arizona.edu. We welcome your comments and suggestions.

9. Brendan Fry, Jack Lee, Nic Smith, Amy Smith, Bohan Li and Tim Secomb contributed to the development of this method.

10. This program is freely available for non-commercial use, provided appropriate acknowledgement is given. Commercial users please contact us before using this program. No assurance is given that it is free of errors and any use is at the user’s risk.

3. GreensV4_GPU: Simulation of solute transport in vascular networks (GPU version)

https://github.com/secomb/GreensV4_GPU

In the Green's function method for simulating solute transport from a network of vessels to a finite volume of tissue, vessels and tissue are treated as distributions of sources of sinks. The method is described in:
Secomb TW, Hsu R, Park EY, Dewhirst MW. 2004 Green's function methods for analysis of oxygen delivery to tissue by microvascular networks. Ann. Biomed. Eng 32, 1519-1529.

Notes

1. This method uses an "infinite-domain solution," in which the network of vessels and the associated tissue domain are effectively embedded in an infinite space with zero net exchange of solute across the tissue domain boundaries. Such an approach has two advantages: it is applicable to tissue domains of arbitrary shape, and it avoids artifacts that can occur when specific boundary conditions, such as the no-flux condition, are imposed.

2. This method can be used for multiple reacting solutes. It allows for diffusible and non-diffusible solutes. Diffusible solutes may be specified as permeating vessel walls or non-permeating. Oxygen is handled differently from other solutes to account for binding by hemoglobin. Intravascular resistance to solute transport can be represented as a function of vessel diameter, as is the case for oxygen. For other solutes, the permeability of the vessel wall to the solute can be specified.

3. The network of vessels is approximated by a set of straight uniform segments located inside a reference cuboidal (box) shape. This cuboid must include all tissue regions associated with the given network. Each segment is then divided into sub-segments with equal length. The midpoint of each sub-segment represents a source. The tissue domain is divided into small subregions, each centered on a tissue node point. The tissue node points form a three dimension matrix. Inside each subregion, the solute reaction rates are assumed to be uniform, and depend on the solute levels at the tissue node.

4. The tissue node points within the tissue domain are determined by the function outboun.cpp, which is called from analyzenet.cpp with argument 1 or 2 to specify the method. Method 1 finds the smallest convex region inside the cuboid which excludes tissue node points that have a distance to the nearest vessel greater than a value specified by the user (Lb in network.dat). Method 2 finds all tissue points within a distance lb of the vessels. The resulting region is the simulated tissue domain, which is embedded in an infinite domain. The whole cuboidal region can be included in the tissue domain by making Lb large enough.

5. We supply several sample problems in the sub-directories. For input file formatting, please see the files in these directories. These files contain further explanatory comments.

6. Input files must be placed in the same working directory where the C code resides. They can be copied from the folders containing sample problems. The following input files are used:
Network.dat This file specifies the network structure, the flow rate and hematocrit of all segments and the PO2 and solute concentrations of boundary nodes. (Note that these solute concentrations are used only for inflow nodes.)
SoluteParams.dat This file gives solute transport and reaction parameters. Oxygen is handled differently than other solutes to account for binding by hemoglobin. Other solutes are assumed to be carried in solution and not to bind to blood components. This file contains oxygen transport parameters, which are needed only if the “oxygen” parameter is set to 1.
ContourParams.dat At each time point, the program generates a postscript file showing the vessel network projected onto a single plane and the solute contours on that plane. If there are multiple solutes, the plots are generated for each plot as separate pages within the postscript file. The position and orientation of the plane are specified by ContourParams.dat, giving the coordinates of three corners of the plane. Also, the contour levels are specified in this file.
IntravascRes.dat Specifies intravascular resistance to solute transport from blood to tissue. For oxygen, this depends on the vessel diameter. For other solutes, a wall permeability is specified by the file.
VaryParams.dat The program provides the capability to specify multiple runs with different parameter values. In version 4, an optional VaryParams.dat file can be included. The following parameters can be varied:
q0fac, diff[isp], tissparam[i][isp] - specify actual values
solutefac[isp], intravascfac[isp] - specify multiplicative factors
A run is performed for each specified set of parameters. An example is included in the “Brain” folder. Note that the values specified in VaryParams.dat override the values given in the other input data files.
tissrate.cpp.dat This is a segment of C code supplied by the user that gives the rates of production mtiss[isp] of solute number isp in the tissue as a function of the solute concentrations c[1], c[2], …, c[nsp]. Also, the derivatives mptiss[isp] of mtiss[isp] with respect to c[isp] must be specified in this code. Caution: If this file is modified, the project must be rebuilt otherwise the old version will remain in the compiled code.
postgreens.cpp.dat This user-supplied c code specifies computation of dependent variables from the results of greens. For example, concentrations of cytotoxic drugs can be used to compute cellular survival fractions at each point in the tissue.
PostGreensParams.dat This file specifies parameters that are used in postgreens.cpp.

7. The program generates several output files giving results in numerical form. It generates a folder called "Current" in the working folder (if it does not already exist), and generates postscript files showing the vessel network and the contours of each solute, in the "Current" folder. The contouring routine produces a color-shaded contour plot. It includes procedures to consolidate polygon of the same color, so as to reduce the size of the resulting output postscript files. The network is superimposed on the contour plot, and is plotted in order of increasing z with vessels outlined to give a pseudo three-dimensional effect. It can be viewed using a postscript viewer, such as GSview https://www.ghostscript.com/download/gsdnld.html.

8. Other output files: **summary.out** - Summary of runs; useful when VaryParams.dat is used. **VesselSources.out, VesselLevels.out, TissueSources.out, TissueLevels.out** - Data on solute levels and source strengths at vessel points and at tissue points.
greens.exelem, greens.exnode, greens.com.txt - Files used to obtain 3D visualizations of the network using CMGUI. When CMGUI is started, use File - Open - com file, select greens.com.txt and hit "All" for the visualization. For details of cmgui, see: http://sourceforge.net/projects/cmiss/files/cmgui/cmgui-wx-2.8.0/

9. We have tested this package using Microsoft Visual C++ 2017 under Windows. For instructions on setting up a new project with this compiler, please see NewProject.txt. For error reporting and suggestions please contact Dr. Timothy W. Secomb, email secomb@arizona.edu. We welcome your comments and suggestions.

10. This program is freely available for non-commercial use, provided appropriate acknowledgement is given. Commercial users please contact us before using this program. No assurance is given that it is free of errors and any use is at the user's risk.

Version 4 changes

1. Method to vary blood flow rates throughout network. In versions 3 and below, the parameter q0 was included (SoluteParams.dat, line 10) to give the total inflow to the network. In version 4, a parameter q0fac is defined (SoluteParams.dat, line 10). When the program is run, all segment flows specified in Network.dat are multiplied by q0fac. This change is NOT compatible with SoluteParams.dat files used for versions 3 and below.

2. Number of tissue parameters per solute. In versions 3 and below, three tissue parameters at most could be defined per solute. In version 4, the number of parameters is defined as the second entry in line 11 of SoluteParams.dat. This change is NOT compatible with SoluteParams.dat files used for versions 3 and below.

3. Capability to specify multiple runs with different parameter values has been added. See above.

4. Capability for 3D visualization of networks using CMGUI has been added. See above.

5. Capability has been added to compute dependent quantities after greens has run. See above.

6. Summary output file from multiple runs (summary.out) has been added.

7. Output files are formatted differently. TissueLevels.dat, TissueSources.dat, VesselLevels.dat, VesselSources.dat now have one line per tissue point or vessel point, for ease of importing into spreadsheets.

8. Previous combined output file (GreensRes.out) is no longer generated.

9. The capability to restart a run has been removed (was not functional).

10. All output files and a copy of input files are placed in a folder called "Current."

11. The algorithm for non-diffusible solutes has been improved by using reaction rates based on updated levels of other solutes at each iteration of Newton method.

12. Error bounds have been modified to use relative errors instead of absolute errors.

4. AngioAdaptRetina: Simulation of angiogenesis, remodeling and pruning in the retina

https://github.com/secomb/AngioAdaptRetina

See Alberding, J.P. and Secomb, T.W. Simulation of angiogenesis in three dimensions: Development of the retinal circulation. Bulletin of Mathematical Biology 85:27 (2023) (26 pages)

See also Alberding JP, Secomb TW. Simulation of angiogenesis in three dimensions: Application to cerebral cortex. PLoS Comput Biol. 17:e1009164 (2021). doi: 10.1371/journal.pcbi.1009164.

See also Secomb, T.W., Alberding, J.P., Hsu, R., Dewhirst, M.W. and Pries, A.R. Angiogenesis: an adaptive biological patterning problem. PLoS Computational Biology 9:e1002983. (2013) doi:10.1371/journal.pcbi.1002983

See https://github.com/secomb/FlowEstimateV1 for more details on calculation of blood flow in the network.

See https://github.com/secomb/GreensV4 for more details on Green's function method to calculate spatial distributions of oxygen and growth factor in tissue.

Input files

Network.dat This file specifies the network structure, the flow rate and hematocrit of all segments and the PO2 and solute concentrations of boundary nodes. (Note that these solute concentrations are used only for inflow nodes.):
SoluteParams.dat This file gives solute transport and reaction parameters. Oxygen is handled differently than other solutes to account for binding by hemoglobin. Other solutes are assumed to be carried in solution and not to bind to blood components. This file contains oxygen transport parameters, which are needed only if the "oxygen" parameter is set to 1.:
ContourParams.dat At each time point, the program generates a postscript file showing the vessel network projected onto a single plane and the solute contours on that plane. The position and orientation of the plane are specified by ContourParams.dat, giving the coordinates of three corners of the plane. Also, the contour levels are specified.:
IntravascRes.dat Specifies intravascular resistance to solute transport from blood to tissue. For oxygen, this depends on the vessel diameter. For other solutes, a wall permeability is specified by the file.:
RheolParams.dat This file gives parameters for equations to describe the apparent viscosity of blood in microvessels in vivo as a function of vessel diameter and discharge hematocrit, and the partition of hematocrit in diverging bifurcations, as a function of the flow rates in the branches, the diameters of the branches, and the hematocrit arriving at the parent vessel. Set varyviscosity = 1 to get diameterdependent viscosity. Set phaseseparation = 1 to compute phase separation in diverging bifurcations. This can lead to nonconvergence of the method in some cases. Also, the number of iterations for linear and nonlinear loops are specified. These may need to be increased for very large networks.:

AngioParams.dat This file gives the values of parameters associated with angiogenesis and vessel migration, including:

- probability of sprout formation:
- maximum number of new sprouts per time step:
- Vmax, um/day rate of growth:
- threshold GF concentration for sprout formation:
- variance, gives the amount of wiggle in direction of new growth:
- diameter of new vessels
- parameters in threshold function for sprouting probability:
- strength of responses to gradients in GF:
- strength of signal from cone detector (guidance to other vessels):
- threshold for movement under tension:
- rate of node movement under tension:
- seed for randomization:
- cutoff radius for cone detector
- cutoff angle for cone detector:
- top of the Inner Nuclear Layer:
- bottom of INL:
- increased GF production in INL, decreased production below INL:
- include hexagonal "astrocyte" guidance, side of hexagon, strength of signal from hexagon:
- include retinal thickening, no. of time steps, amount (micron) per time step:
- thickness of photoreceptor layer, increase in O2 demand in photoreceptor layer: :

AdaptParams.dat This file gives the values of parameters associated with structural adaptation and pruning, including:

- number of adaptation/angiogenesis time steps:
- time step, day:
- parameters for pressureshear relationship:
- overall strength of metabolic signal:
- max. upstream response relative to downstream:
- saturation levels for downstream and upstream responses, um:
- shrinking tendency:
- conducted response decay length:
- diameter threshold for dropout:
- adaptation time scale:

Output files

The program creates a new folder called "Current" if it does not already exist. As the simulation proceeds, files are placed in this folder as follows, where xxx is the current time step number::
Networkxxx.dat The current network file.:
network.exelem and **networkxxx.exnode** These files are used to obtain 3D visualizations of the network using CMGUI. When CMGUI is started, use File: Open: com file, select greens.com.txt and hit “All” for the visualization. For details of cmgui, see:
http://sourceforge.net/projects/cmiss/files/cmgui/cmguiwx2.8.0/:

AdaptSignalsxxx.ps This file gives a graphical representation of the current signals for diameter change, as a function of intravascular pressure. The color code is: red: shear stress; green: pressure; blue: shrinking tendency; purple: upstream conducted response; olive: downstream convected response; black: total signal.:
ContourO2GF000.ps This file shows a map of the current network projected onto the specified contour plane. Oxygen levels are shown by colored contours on the plane, and growth factor levels above threshold are indicated by diagonal hatching.:

At the end of the simulation, multiple numerical and graphical files are produced to show the final state of the network, such as histograms of vessel flow rate, flow velocity and shear stress, and vessel and tissue oxygen levels.

This code is free to use at your own risk. Feedback and/or acknowledgement are appreciated. Email secomb@arizona.edu.:

Note: This code makes use of nrutil.h and nrutil.cpp as placed in the public domain by Numerical Recipes at http://apps.nrbook.com/c/index.html.
